# Non-response to preoperative chemotherapy is a contraindication to hepatectomy plus radiofrequency ablation in patients with colorectal liver metastases

**DOI:** 10.18632/oncotarget.20647

**Published:** 2017-09-05

**Authors:** Rui Mao, Jian-Jun Zhao, Hong Zhao, Ye-Fan Zhang, Xin-Yu Bi, Zhi-Yu Li, Jian-Guo Zhou, Xiao-Long Wu, Chen Xiao, Jian-Qiang Cai

**Affiliations:** ^1^ Department of Hepatobiliary Surgery, Cancer Hospital, CAMS, Beijing 100021, P.R.China

**Keywords:** colorectal liver metastases, hepatectomy, radiofrequency ablation, preoperative chemotherapy, response

## Abstract

The long-term outcome of 228 patients with colorectal liver metastases (CRLM) who underwent preoperative chemotherapy followed by hepatectomy ± RFA were retrospectively analyzed. Stratified by chemotherapy response, patients were divided into responding (n=129) and non-responding groups (n=99). Patients who underwent hepatectomy-RFA had a greater number of metastases (median of 4 vs. 2, p=0.000), a higher incidence of bilobar involvement (66.7% vs. 49.1%, p=0.014) and longer chemotherapy cycles (median of 6 vs. 4, p=0.000). In the responding group, the median overall survival (OS) and recurrence free survival (RFS) of hepatectomy-RFA and the hepatectomy alone subgroups were comparable (38.6 months vs. 43.2 months, p=0.824; 8.2 months vs. 11.4 months, p=0.623). In the non-responding group, the median OS and RFS of patients treated with hepatectomy-RFA were significantly shorter (18.5 months vs. 34.2 months, p=0.000; 5.1 months vs. 5.9 months, p=0.002). RFA was identified as the unfavorable independent factor for both OS (HR=3.60, 95%CI=1.81-7.16, p=0.039) and RFS (HR=1.70, 95%CI=1.00-2.86, p=0.048) in non-responsive patients. Local recurrence rate after hepatectomy-RFA was higher in the non-responding group (48.1% vs. 23.6%, p=0.018). Non-response to preoperative chemotherapy may be a contraindication to hepatectomy-RFA in patients with CRLM.

## INTRODUCTION

Liver is the most frequent site for metastasis from colorectal cancer, with more than 50% of patients developing hepatic metastases during the course of the disease [1, 2]. Liver resection combined with modern chemotherapy is considered the treatment of choice for patients with CRLM [3, 4]. For CRLM judged to be resectable, preoperative chemotherapy is considered a standard of care in most Western countries [5]. For patients with CRLM not eligible for resection at diagnosis due to risk of subsequent liver insufficiency or maldistribution of hepatic metastases, chemotherapy can convert the unresectable or borderline resectable cases into resectable disease [6-10]. Besides, a correlation between response to preoperative chemotherapy and long-term outcome has also been confirmed [11].

RFA, as a complement of hepatic resection, allows surgeons to ablate small lesions while resecting large ones, aiming to preserve adequate liver parenchyma. However, the therapeutic efficacy of hepatectomy-RFA in the treatment of CRLM remains controversial because of relatively high intrahepatic recurrence rate and unfavorable survival [12-14]. It should be noted that patients treated with resection-RFA or resection alone were different for baseline tumor characteristics [15]. It is hard to determine whether RFA impacts survival negatively because of a more aggressive disease biology that makes treatment with resection alone impossible. Therefore, it is more reasonable to identify a group of patients who will benefit from this treatment modality.

Previous studies have demonstrated that good long-term outcomes can be achieved by a combination of prior chemotherapy and RFA with or without liver resection in patients with unresectable CRLM [16-18]. We postulate chemotherapy response could also predict therapeutic effect of this treatment. However, the proportions of patients receiving preoperative chemotherapy vary among studies, and the impact of response on outcome is seldom assessed. In current study, we compared the outcome of patients treated with hepatic resection alone or resection-RFA, grouped by response to preoperative chemotherapy. The clinical efficacy of hepatectomy-RFA was comparable to hepatectomy alone in patients who responded to preoperative chemothearpy. Whereas, the long-term results of non-responding patients who underwent hepatectomy-RFA were significantly worse.

## RESULTS

### Patient characteristics

The clinicopathologic features of the patients in the study are displayed in Table 1. The study population comprised 228 patients (145 men and 83 women; median age, 54 years). Most patients (83.8%) developed synchronous liver metastases. 75.9% of the patients had more than one metastases, with a median of 3 lesions, and a maximum of 9 lesions. The median diameter of the largest lesion was 2.8cm, and 49.6% of patients had lesions larger than 3 cm. Bilobar distribution of metastases was observed in 54.4% of the patients. Preoperative chemotherapy regimens included oxaliplatin-based (70.2%), irinotecan-based (17.1%), oxaliplatin and irinotecan-based (12.7%). Biological agents were included in preoperative regimens for 28.5% of the patients. The median number of preoperative chemotherapy cycles was 5, with 46 patients (20.2%) receiving at least 8 cycles. PR, SD and PD to preoperative chemotherapy were observed in 129 (56.3%), 68 (29.8%) and 31 (13.6%) patients respectively. A total of 69 patients (30.3%) underwent hepatectomy in combination with RFA; 159 patients (69.7%) underwent hepatectomy alone. The total number of resected lesions were 570, averaging 2.5 per patient, with 148 lesions treated by RFA (2.1 per patients). The median diameter of resected and abalted lesions were 2.6 cm and 2.1 cm, respectively. 81 patients (35.5%) had margin invasion, and major complications were reported in 39 patients (17.1%). Postoperative chemotherapy for CRLM was administered to 196 patients (86.0%).

**Table 1:** Clinicopathologic characteristics of patients with CRLM sorted by treatment

	All Patients n=228	Resection alone n=159	Resection+RFA n=69	p
Male sex, n (%)	145(63.6)	100(62.9)	45(65.2)	0.738
Age, (range)	54(28-79)	54(28-79)	55(29-72)	0.570
Age ≥60, n (%)	71(31.1)	50(38.8)	21(30.4)	0.88
Preoperative CEA, (range), ng/ml	7.6 (0.8-1503.0)	7.0(0.8-1503.0)	9.3(1.3-147.0)	0.527
Preoperative CEA ≥10 ng/ml, n (%)	97(42.5)	65(40.9)	32(46.3)	0.441
Primary site, n (%)				
Colon	137(60.1)	86(54.1)	41(59.4)	0.456
Left hemicolon	195(85.5)	138(86.8)	57(82.6)	0.409
Synchronous metastasis, n (%)	191(83.8)	131(82.4)	60(87.0)	0.390
T3-4, n (%)	207(90.8)	146(91.8)	61(88.4)	0.412
Positive lymph nodes, n (%)	154(67.5)	108(67.9)	46(66.7)	0.852
Lymphovascular invasion	79(34.6)	56(35.2)	23(33.3)	0.783
Perineural invasion	80(35.1)	60(37.7)	20(29.0)	0.203
KRAS mutation, n (%)*	56(40.6)	33(39.8)	23(41.8)	0.809
≥4 hepatic metastases, n (%)	99(43.4)	51(32.1)	48(69.6)	0.000
Number of metastases, (range)	3(1-9)	2(1-8)	4(2-9)	0.000
Number of ablated lesions, (range)	-	-	2(1-4)	-
Number of resected lesions, (range)	2(1-8)	2(1-8)	2(1-5)	0.053
Bilobar distribution, n (%)	124(54.4)	78(49.1)	46(66.7)	0.014
Biggest metastatic volume in cms, (range)	2.8(0.5-10)	2.8(0.5-10)	3(0.5-8.5)	0.338
Largest diameter ≥3 cm, n (%)	113(49.6)	76(47.8)	37(53.6)	0.419
Preoperative chemotherapy regimen, n (%)				
oxaliplatin	160(70.2)	107(67.3)	53(76.8)	0.149
irinotecan	39(17.1)	24(15.1)	15(21.7)	0.221
Oxaliplatin+irinotecan	29(12.7)	22(13.8)	7(10.1)	0.442
Biological agents, n (%)	65(28.5)	42(26.4)	23(33.3)	0.288
Preoperative chemotherapy cycles, (range)	5 (2-22)	4(2-14)	6(2-22)	0.000
Preoperative chemotherapy cycles ≥8, n (%)	46(20.2)	22(13.8)	24(34.8)	0.000
Response to chemotherapy, n (%)	129(56.3)	87(54.7)	42(60.9)	0.389
Surgical procedure				
Nonanatomical resection	171(75.0)	115(72.3)	56(81.1)	0.157
Anatomical resection	25(11.0)	21(13.2)	4(5.8)	0.1
Anatomical+nonanatomical resection	32(14.0)	23(14.5)	9(13.0)	0.776
Positive surgical margins, n (%)	81(35.5)	55(34.6)	26(37.7)	0.654
Postoperative chemotherapy, n (%)	196(86.0)	135(84.8)	61(88.4)	0.485
Major complications, n (%)	39(17.1)	19(11.9)	20(29.0)	0.002

RFA=Radiofrequency Ablation. CRLM=Colorectal Liver metastases. CEA=Carcino-embryonic antigen. KRAS=Kirsten rat sarcoma viral oncogene.

* KRAS status was available in 138 patients.

Patients who underwent hepatectomy-RFA had a greater number of total liver lesions (median of 4 vs. 2, p=0.000). Bilobar distribution of liver lesions was also more common in patients who underwent hepatectomy-RFA (66.7% vs. 49.1%, p=0.014). The median cycle of preoperative in hepatectomy-RFA cohort was six, compared with the four cycles in hepatectomy alone group (p=0.000). The proportion of patients who developed major complications were 29.0% and 11.9% after hepatectomy-RFA or hepatectomy alone, respectively (p=0.002).

### Overall survival

The median follow-up of the entire study population was 32 months. A total of 98 patients (42.9%) died during follow-up. Median survival was 35.7 months. The 1-, 3-, and 5-year OS rates were 93.3%, 50.5%, and 20.8%, respectively. According to response to preoperative chemotherapy, the included patients were divided into responding group (PR, n=129) and non-responding group (SD/PD, n=99). Risk factors for decreased OS are displayed in Table 2.

**Table 2:** Multivariate analysis of clinicopathologic factors associated with overall survival in patients with CRLM according to the response to preoperative chemotherapy

	Median OS (months)	5-year OS (%)	HR (95% CI)	multivariate p
All patients (n=228)				
≥4 hepatic metastases	29.7	11.3	1.54 (1.00-2.35)	0.008
Bilobar distribution	31.4	9.4	2.32 (1.51-3.59)	0.000
Non-responsive to chemotherapy	31.6	11.1	1.89 (1.25-2.86)	0.003
Positive lymph nodes	33.8	16.0	1.66(1.01-2.71)	0.44
Major complication	24.1	12.4	2.52(1.56-4.06)	0.000
Responding group (n=129)				
Preoperative chemotherapy cycles ≥8	33.8	0	3.26(1.61-6.61)	0.01
Major complication	24.1	13.2	3.37(1.56-7.32)	0.002
Non-responding group (n=99)				
Intraoperative RFA	18.5	0	3.60 (1.81-7.16)	0.039
Bilobar distribution	20.7	0	2.13(1.20-3.79)	0.01

RFA=Radiofrequency Ablation. CRLM=Colorectal Liver Metastases. OS=Overall Survival.

HR=Hazard Ratio. CI=Confidence Interval.

**Figure 1:**
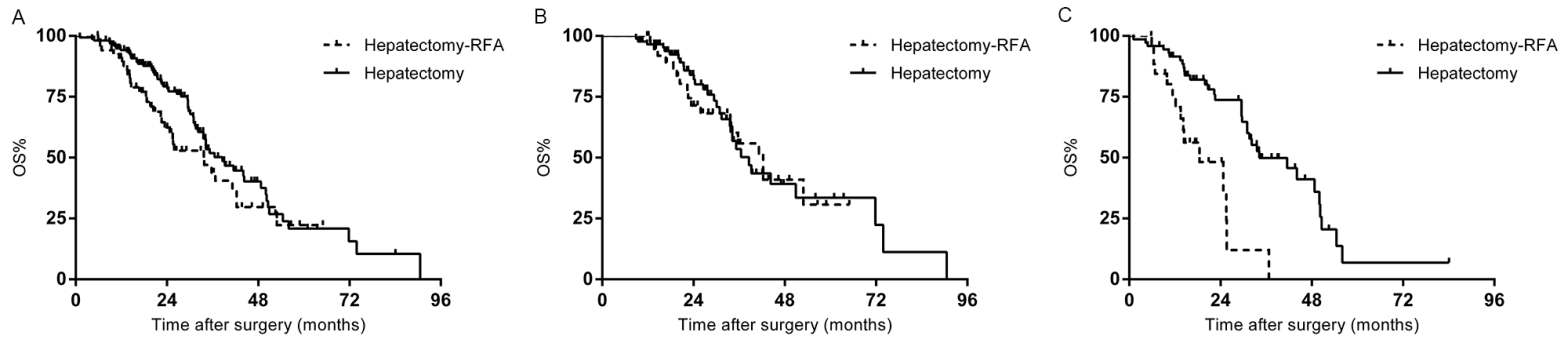
**(A)** Overall survival (OS) of the whole study population stratified by hepatectomy with or without RFA. **(B)** OS of the responding group stratified by hepatectomy with or without RFA. **(C)** OS of the non-responding group stratified by hepatectomy with or without RFA.

For all included patients, there was no significant difference in survival rate between the patients who underwent hepatectomy-RFA and those treated by hepatectomy alone at 5 years (22.3% vs. 20.9%, p=0.096) (Figure 1A). Preoperative chemotherapy cycles (p=0.001), regimens (p=0.006), and responses (p=0.005); primary tumor lymph node metastases (p=0.01); liver lesion numbers (p=0.001), distribution (p=0.001), and diameters (p=0.057); resection margin status (p=0.015), and major complications (p=0.000) were associated with OS in univariate analysis. In multivariate analysis, ≥4 hepatic metastases (p=0.008), bilobar distribution (p=0.000), non-responsive to chemotherapy (p=0.003), lymph node metastases (p=0.44) and major complications (p=0.000) predicted decreased OS for the whole cohort of patients.

The median follow-up of the responding group (129 patients) was 31.6 months. 5-year survival rate of hepatectomy-RFA and the hepatectomy alone subgroups were comparable (30.7% vs. 33.6%, p=0.824) (Figure 1B). Multivariate analysis revealed that prolonged chemotherapy (p=0.01), and major complications (p=0.002) remained as significant predictive factors for unfavorable survival.

The median follow-up of the non-responding group (99 patients) was 32 months. The median survival time for 27 patients who received hepatectomy-RFA was 18.5 months, significantly shorter than 34.2 months observed in those treated with hepatectomy alone (p=0.000) (Figure 1C). In multivariate analysis, intraoperative RFA (p=0.039) and bilobar distribution (p=0.001) were the predictors of worse OS.

### Recurrence-free survival

**Figure 2:**
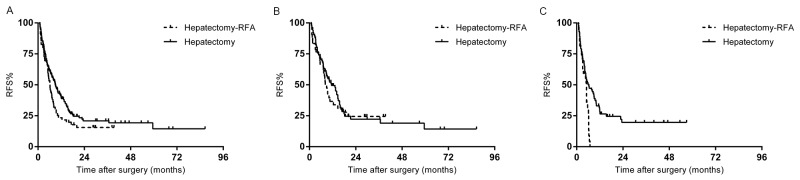
**(A)** Recurrence free survival (RFS) of the whole study population stratified by hepatectomy with or without RFA. **(B)** RFS of the responding group stratified by hepatectomy with or without RFA. **(C).** RFS of the non-responding group stratified by hepatectomy with or without RFA.

Risk factors for decreased RFS are displayed in Table 3. For the entire study population, the median recurrence-free survival was 7.7 months. The 1-, 3-, and 5-year RFS rates were 33.7%, 19.4%, and 13.6%, respectively. Patients who underwent hepatectomy alone had a longer median RFS than patients who underwent hepatectomy-RFA (9.0 months vs. 6.2 months, p=0.05) (Figure 2A). Preoperative chemotherapy cycles (p=0.001), regimens (p=0.006), and responses (p=0.005); primary tumor lymph node metastases (p=0.011), and perinueral invasion (p=0.008); liver lesion numbers (p=0.000), and distribution (p=0.001); surgical procedure (p=0.024), resection margin status (p=0.001), and major complications (p=0.000) were associated with RFS in univariate analysis. In multivariate analysis, predictive factors for decreased RFS were ≥4 hepatic metastases (p=0.000), non-responsive to chemotherapy (p=0.002), and major complications (p=0.015).

**Table 3:** Multivariate analysis of clinicopathologic factors associated with recurrence free survival in patients with CLM according to the response to preoperative chemotherapy

	Median RFS (months)	3-year RFS (%)	HR (95% CI)	multivariate p
All patients (n=228)				
≥4 hepatic metastases	5.6	11.3	1.87(1.35-2.58)	0.000
Non-responsive to chemotherapy	5.1	14.9	1.67(1.21-2.29)	0.002
Major complication	4.9	10.1	1.63 (1.10-2.41)	0.015
Responding group (n=129)				
Preoperative chemotherapy cycles ≥8	6.4	14.8	3.37(1.56-7.32)	0.002
Bilobar distribution	7.4	15.2	1.84(1.18-2.88)	0.007
Non-responding group (n=99)				
Intraoperative RFA	5.1	4.4	1.70 (1.00-2.86)	0.048
≥4 hepatic metastases	4.2	0	1.87(1.35-2.58)	0.000

RFA=Radiofrequency Ablation. CRLM=Colorectal Liver Metastases. RFS=Recurrence Free Survival. HR=Hazard Ratio. CI=Confidence Interval.

In the responding group (129 patients), the median RFS of patients who underwent hepatectomy-RFA was 8.2 months, not significantly different from the 11.4 months after treatment of hepatectomy alone (P=0.623) (Figure 2B). Multivariate analysis demonstrated that bilobar distribution (p=0.007), and prolonged chemotherapy (p=0.002) were independent predictors for recurrence.

In the non-responding group (99 patients), all patients who received hepatectomy-RFA experienced recurrence within one year of operation, while the 3-year RFS rate for patients after hepatectomy alone was 19.7% (p=0.002) (Figure 2C). In multivariate analysis, the significant predictors of higher recurrence was intraoperative RFA (p= 0.048), and ≥4 hepatic metastases (p=0.000).

### Patients who underwent hepatectomy-RFA

**Figure 3:**
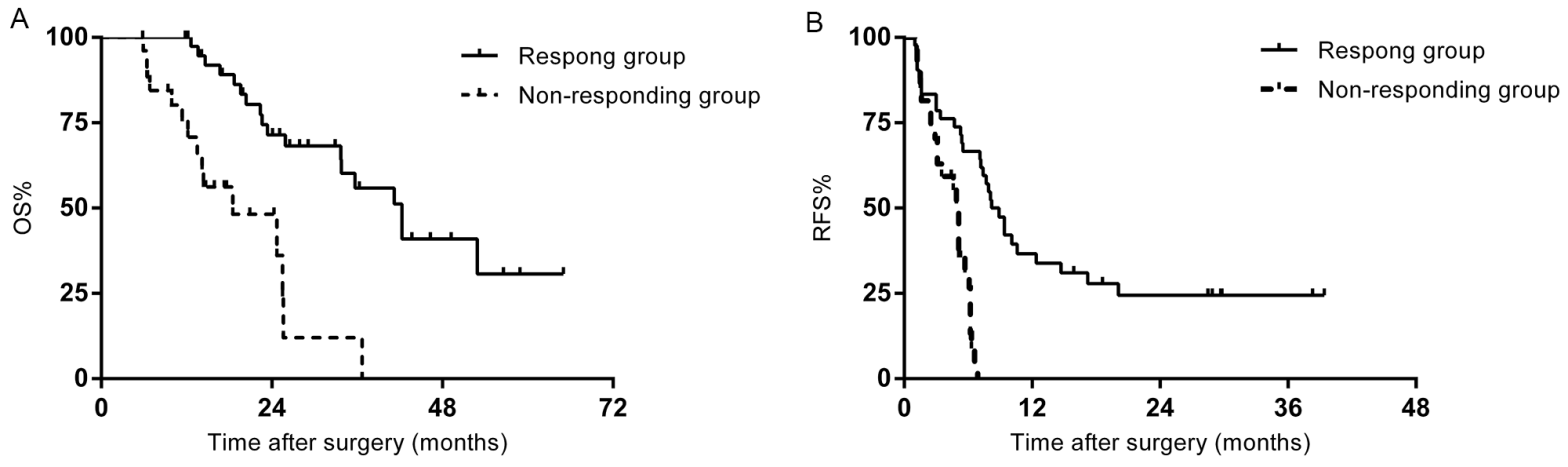
**(A)** OS of the patients who underwent hepatectomy-RFA stratified by chemotherapy response. **(B)** RFS of the patients who underwent hepatectomy-RFA stratified by chemotherapy response.

A separate analysis was performed in sixty-nine patients who underwent hepatectomy-RFA. The median survival of the responding group was 42.3 months, significantly longer than the 18.5 months in the non-responding group (p=0.000) (Figure 3A). A significant difference was also found in RFS between the two groups (8.2 months vs. 5.1 months, p=0.000) (Figure 3B). Multivariate analysis demonstrated that non-responsive to chemotherapy (p=0.000) and ≥4 hepatic metastases (p=0.008) were independent predictors for unfavorable survival. The predictive factors for recurrence were non-responsive to chemotherapy (p=0.013), and major complications (p=0.000).

### Patterns of recurrence

A total of 167 (73.2%) patients experienced recurrence, including intrahepatic, extrahepatic, and both intra- and extrahepatic recurrences in 128 (56.1%), 11 (4.8%) and 28 (12.3%) patients, respectively. Differences in recurrence patterns among the treatment groups is shown in Table 4. The overall recurrence rate after hepatectomy-RFA and hepatectomy alone were 76.8% and 71.7%, respectively (p=0.423). The dominant pattern of recurrence was intrahepatic (63.8% vs. 52.8%, p=0.126); local recurrence rate at the ablation site/resection margin was documented in 31.9% of patients who underwent hepatectomy-RFA, compared with 18.2% after hepatecomy alone (p=0.023). The proportion of patients in two treatment groups found to have extrahepatic or both intra- and extrahepatic recurrences was similar.

**Table 4:** Patterns of recurrence sorted by treatment

	Hepatectomy+RFA n=69 (%)	Hepatectomy n=159 (%)	P
Overall recurrence	53(76.8)	114(71.7)	0.423
Intrahepatic recurrence	44(63.8)	84(52.8)	0.126
Ablation site	12(17.4)	-	
Resection margin	10(14.5)	29(18.2)	0.49
Local recurrence	22(31.9)	29(18.2)	0.023
New metastases	22(31.9)	55(34.6)	0.691
Intra+Extrahepatic recurrence	5(7.2)	23(14.5)	0.127
Extrahepatic recurrence	4(5.8)	7(4.4)	0.738

RFA=Radiofrequency Ablation. Local recurrence=ablation site and resection margin recurrence

Recurrence pattern after surgery in the responding and non-responding groups is shown in Table 5. In the non-responding group, recurrence of any kind occurred more frequently after hepatectomy-RFA (92.6%), versus that after resection alone (76.4%, p=0.068). Both intrahepatic and local recurrence rates after hepatectomy-RFA were significantly higher than those in patients treated with resection alone (77.8% vs. 55.6%, p=0.043; 48.1% vs. 23.6%, p=0.018). Rate of recurrence at the ablation site was comparable to that at the margin after resection alone, whether in the entire group (17.4% vs. 18.2%, p=0.868) or both subgroups separately (responding group: 11.9% vs. 13.8%, p=0.766; non-responding group: 25.9% vs. 23.6%, p=0.8111). Significant differences in recurrence patterns between two treatment modalities were not observed in the responding group.

**Table 5:** Patterns of recurrence according to the response to preoperative chemotherapy

	Responding group			Non-responding group		
	Hepatectomy+RFA n=42 (%)	Hepatectomy n=87 (%)	p	Hepatectomy+RFA n=27 (%)	Hepatectomy n=72 (%)	p
Overall recurrence	28(66.7)	59(67.8)	0.896	25(92.6)	55(76.4)	0.068
Intrahepatic recurrence	23(54.8)	44(50.6)	0.656	21(77.8)	40(55.6)	0.043
Ablation site	5(11.9)	-	-	7(25.9)	-	-
Resection margin	4(9.5)	12(13.8)	0.491	6(22.2)	17(23.6)	1.0
Local recurrence	9(21.4)	12(13.8)	0.271	13(48.1)	17(23.6)	0.018
New metastases	14(33.3)	32(36.8)	0.515	8(29.6)	23(31.9)	0.825
Intra+Extrahepatic recurrence	3(7.1)	11(12.6)	0.523	2(7.4)	12(16.7)	0.339
Extrahepatic recurrence	2(4.8)	4(4.6)	1.0	2(7.4)	3(4.2)	0.612

RFA=Radiofrequency Ablation. Local recurrence=ablation site and resection margin recurrence

## DISCUSSION

Improvements in preoperative chemotherapy and combination hepatectomy with RFA allow radical treatment for an increased number of patients with CRLM. This study suggested that the benefits associated with this treatment modality were strongly impacted by response to chemotherapy.

The median survival of the entire study population was 35.7 months in present study, inferior to the reported survival of 40.5–48.2 months after conversion therapy [19-21]. We attributed this to the relatively lower response rate (56.3%) compared to a mean value of 74% (range 60–100%) reported in previous studies [20]; and the variable definition of resectability. The median RFS after conversion therapy was 7.7 months, which is in line with previous results (3.2-10.6 months) [19-21], reaffirming the finding of relatively short RFS and a prolonged OS after conversion therapy followed by surgery.

Objective evaluation of hepatectomy-RFA compared to resection alone is difficult. Indeed, patients who underwent hepatectomy-RFA were characterized by greater number and more widespread distribution of liver lesions in current study. Besides, these patients seem to be less sensitive to chemotherapy (prolonged chemotherapy was required in 34.8% of patients). Recently, Imai et al used propensity score matching to overcome imbalance of background characteristics [22]. After matching, overall and disease free survival in hepatectomy-RFA group were not different from those of patients treated with hepatectomy alone. The results justified the adjunct use of RFA in hepatectomy for selected patients. Whereas, detailed indications and contraindications for this treatment is still unclear.

RFA is usually regarded as an alternative therapeutic option with poor disease control for unresectable CRLM [23-24]. Indeed, local recurrence rate following hepatectomy-RFA was significantly higher in the entire study population (31.9% vs. 18.2%, p=0.023) and non-responding group (48.1% vs. 23.6%, p=0.018). Nevertheless, this was not observed in chemotherapy-responsive patients (21.4% vs. 13.8%, p=0.271). Although patients who underwent hepatectomy-RFA were characterized by a larger tumor burden, the responding group showed similar long-term survival after two treatment modalities. Besides, rate of recurrence at the ablation site was comparable to that at the margin after resection alone, which is similar to the result of a study from Eltawil et al [25]. The authors reviewed 24 patients who underwent hepatectomy-RFA for CRLM and demonstrated an ablation site local recurrence rate of 9.5%, which compared favorably among patients who underwent hepatectomy alone (20%), suggesting that RFA is not associated with an excess ablation site recurrence. Prospective survival data for patients treated with chemotherapy-RFA also comfirmed our results. The CLOCC trial (chemotherapy alone versus chemotherapy + RFA) reported a 3-year PFS of 27.6% and OS at 30 months of 61.7% in the RFA arm [16]. ARF 2003 (chemotherapy + RFA + surgery) found a 3-year PFS of 10% and 30 months OS of 74% [17]. These results were comparable to those of patients who underwent hepatectomy-RFA in the responding group (3-year RFS: 24.4%, 30 months OS:68.2%). Therefore, hepatectomy-RFA should be used for those needed if there is radiological response to prior systemic therapy.

Several studies have demonstrated that progression while preoperative chemotherapy is not an absolute contraindication to liver resection for patients whose disease remains resectable [26-27]. The major concern is the treatment of patients with CRLM whose disease remains unresectable after preoperative chemotherapy. Is liver resection in combination with intraoperative RFA indicated, or should further chemotherapy regimens be planned? Given the low objective response rates to second-line chemotherapy [28-30] and the lack of other effective treatment options for patients with no response to chemotherapy, rescue surgery consist of hepatectomy and RFA was sometimes introduced as an alternative therapeutic option in our hospital. However, whether this treatment modality could work as effectively as hepatectomy alone in non-responsive patients is largely unknown. To our knowledge, few studies have focused on this topic. Our results suggested that hepatectomy-RFA provided significantly worse outcome with 3-year OS rate of 12%, and 3-year RFS rate of 4.4% in patients with downstaging not achieved. This long term result even seems inferior to that of palliative chemotherapy [31-32], which seem to be a result of high major complications rate (29%) and overall recurrence rate (92.6%) following hepatectomy-RFA. Even though one might argue the outcome may be a result of a selection bias, intraoperative RFA showed an independent association with decreased OS and RFS after adjusting for other clinical and pathological factors.

Patients undergoing hepatectomy-RFA mostly had worse tumor biology than those undergoing hepatectomy alone. For responding group, hepatectomy-RFA can make patients with high tumor burden achieve a long-term outcome comparable to that of patients with favorable tumor biology. However, hepatectomy-RFA does not add any value for chemo-resistant patients, which may be attributed to the limited potential for postoperative systemic chemotherapy to cure residual micro-metastases left behind after surgery in this cohort of patients. Indeed, prolonged preoperative chemotherapy required for downstaging was associated with decreased RFS and OS in the responding group, suggesting significant effects of the extent of chemo-sensitivity on reducing recurrence and prolonging survival. Sasaki et al [33] recently performed a retrospective study of 485 patients who underwent curative hepatectomy and identified primary tumor nodal metastases, KRAS mutation, and preoperative high CEA related to survival. For patients who underwent hepatectomy-RFA, those with no or one risk factor had a 5-year OS rate similar to patients treated with resection alone. In contrast, patients with two or more risk factors had a much worse prognosis. The findings imply that patients who would benefit the most from hepatectomy-RFA could be selected using tumor biology related factors, among which chemotherapy response may play an important role.

This study had some limitations. First, it was a retrospective study with relatively small sample size. Second, operations were performed by different surgeons, with their own criteria regarding patients selection for resection or hepatectomy-RFA. What’s more, some lesions judged to be unresectable in the past may now be treated by resection alone, with improved surgical techniques and skills, which may result in selection bias. Furthermore, only patients with no extrahepatic disease were eligible for the current study and these represent only a proportion of patients with CRLM. Last but not least, preoperative chemotherapy regimen was not standardized and this is also a major source of bias.

## MATERIALS AND METHODS

### Inclusion criteria

Patients who underwent surgery for CRLM at the Department of hepatobiliary surgery, Cancer Hospital, Chinese Academy of Medical Sciences between January 1, 2004, and December 31, 2015, were identified from our prospective institutional database. In these patients, patients who met the following criteria were considered for further analysis. Inclusion criteria were: (1) histologically proven colorectal adenocarcinoma liver metastases; (2) preoperative chemotherapy was given, followed by hepatectomy with or without intraoperative RFA for curative intents. Excluion criteria were: (1) extrahepatic metastases detected on preoperative imaging or during surgery; (2) R2 resection; (3) postoperative deaths (noncancer-related 90-day mortality); or (4) a history of prior hepatectomy for CRLM. In total, 228 patients met the eligibility criteria.

### Perioperative management

All patients were evaluated preoperatively, including carcinoembryonic antigen (CEA) levels; abdominopelvic computed tomography (CT) and magnetic resonance imaging (MRI); and chest radiography or chest CT to determine the disease stage. Preoperative chemotherapy was mainly recommended to patients with initially unresectable liver metastases; or to patients with multiple high-risk factors: synchronous metastases, ≥4 hepatic lesions, primary tumor invading nearby tissues/organs and imaged mesenteric nodal disease. The definitions of unresectability were as follows: multiple liver metastases that required resection of more than 70 % of non-tumor liver for removal of all tumors, tumors invading all three hepatic veins, tumors invading both the left and right branches of the hepatic artery or portal vein, and unresectable extrahepatic metastases. Chemotherapy was composed of a combination of 5-fluorouracil/capecitabine and oxaliplatin/irinotecan with or without bevacizumab and cetuximab. Tumor response was assessed according to the Response Evaluation Criteria In Solid Tumors criteria (RECIST, version 1.1) every two cycles. Complete response (CR) was defined as the disappearance of all signs of the current disease recorded from the start of the treatment. Partial response (PR) was defined as a decrease in the size of the tumor of ∼30%. Stable disease (SD) was defined as no disease progression or regression. Progressive disease (PD) was defined as an increase in the tumor size of >20% during or after treatment. All imaging studies were reviewed by at least two independent radiologists until the final conclusion was drawn. Surgery with curative intent was performed for hepatic lesions considered to be treatable by hepatecomy ± RFA, with the residue liver volume of at least 30%. The decision to undertake surgery for controversial cases was reached by consensus of a multidisciplinary team (MDT) including surgeons, oncologists and radiologists.

During surgery, the peritoneal cavity was inspected to exclude previously undetected extrahepatic lesions. Manual liver palpation and intra-operative ultrasound were used to rule out occult lesions and confirm the number, size and location of the liver metastases. If all the lesions were deemed resectable, hepatectomies alone were undertaken. Neither the number nor the size of metastases excluded any patients from resection. Patients were treated with intraoperative RFA when one hepatic lesion was considered unresectable because of deep location or proximity to major vascular structures, so as to avoid extensive hepatectomy, especially for lesions less than 3 cm. The principle of surgery was to remove all detectable lesions with a tumor-free margin. After resection of the resectable metastases, intraoperative ultrasound was performed to guide placement of the RFA needle into the remaining lesions and then ablation was started. Successful ablation was defined as the complete destruction of the tumor with at least a 1-cm zone of normal liver parenchyma in real-time ultrasound. All specimens were subjected to histological evaluation to confirm the pathological diagnosis, number and size of liver lesions, and the width of the surgical margin. In case of multiple liver metastases, the diameter of the largest lesion was defined as the final size, and the closest margin was recorded. R1 resection was defined with a distance from the metastasis edge to the transection line of less than 1 mm.

Postoperative complications were graded according to the Clavien system, and major complications were defined as any grade III or IV complication. After discharge, adjuvant chemotherapy was recommended to most patients.

### Follow-up

After surgery, patients were followed up at regular intervals. Serum CEA and imaging studies were performed to detect any intrahepatic recurrence or distant metastases. The first follow-up occurred one month post-surgery, with subsequent ones every 3 months for up to 2 years, and every 6 months thereafter.

### Statistical analysis

Continuous variables are expressed as the median (range). Continuous and categorical variables were compared by Mann-Whitney U test and Chi square test or Fisher’s exact test respectively. Survival analyses were carried out using the Kaplan-Meier method, with group comparisons by the log rank test. Multivariate models were used for OS and RFS using the Cox proportional hazard method. Clinicopathological factors were included in each model if they achieved a p < 0.1 for significance in univariate regression analysis. Use of RFA was included in each model, irrespective of statistical significance. P < 0.05 was considered to indicate statistical significance. OS was estimated from liver resection to death; RFS was from liver resection to the first documented disease recurrence. Statistical analyses were performed using the SPSS, version 22, Armonk NV, USA.

## CONCLUSIONS

This study showed that hepatectomy-RFA in patients who respond to preoperative chemotherapy is feasible and may be associated with a long-term outcome similar to that after hepatectomy alone. However, the poor results obtained by hepatectomy-RFA in patients with tumor stablization/progression suggest that non-response to preoperative chemotherapy is a contraindication to this treatment in patients with CRLM, and further chemotherapy may be a more reasonable choice for these patients. Chemotherapy response could be a useful tool for selecting patients for hepatectomy-RFA.
